# Gout of hand and wrist: the value of US as compared with DECT

**DOI:** 10.1007/s00330-018-5363-9

**Published:** 2018-04-20

**Authors:** Andrea S. Klauser, Ethan J. Halpern, Sylvia Strobl, Mohamed M. H. Abd Ellah, Johann Gruber, Rosa Bellmann-Weiler, Thomas Auer, Gudrun Feuchtner, Werner Jaschke

**Affiliations:** 1 Radiology Department, Medical University Innsbruck, Anichstrasse 35, 6020 Innsbruck, Austria; 2 Department of Radiology and Urology, Jefferson Prostate Diagnostic and Kimmel Cancer Center, Thomas Jefferson University, 1025 Walnut Street, Suite 1112, Philadelphia, PA 19107 USA; 3 Diagnostic Radiology Department, South Egypt Cancer Institute, Assiut University, Assiut, 71515 Egypt; 4 Department of Internal Medicine II, Medical University Innsbruck, Anichstrasse 35, 6020 Innsbruck, Austria

**Keywords:** Arthritis, gouty, Wrist, Hand, Upper extremity, Tomography, x-ray computed

## Abstract

**Objectives:**

The purpose of this study was to compare findings of ultrasound (US) with dual-energy CT (DECT) in patients presenting with suspected gouty hand and wrist arthritis.

**Methods:**

This prospective study included 180 patients (136 men and 44 women, age range, 31– 94 years; mean age, 65.9 years) with an initial clinical diagnosis of acute gouty arthritis who underwent DECT and US examination. Intra- and extra-articular findings of each modality were tabulated and calculated with DECT as gold standard.

**Results:**

The final diagnosis of gout was positive in 97/180 patients (53.9%) by DECT, an alternative diagnosis confirmed in 83 patients. US showed a sensitivity of 70.1% (extra-articular: 42.5%, *p* < 0.0001; intra-articular: 80.3%, *p* = 0.14) and specificity of 51%. The double contour sign (DCS) was present in 58/61 patients with a positive US study for intra-articular gout (95.1%).

**Conclusions:**

Sensitivity of US for diagnosis of gouty arthritis in hand and wrist is limited, particularly with respect to extra-articular urate deposition. The DCS is the most sensitive sign for the assessment of gouty hand and wrist arthritis by US.

**Key points:**

*• Sensitivity of US for diagnosis of gouty arthritis in hand and wrist is limited, particularly with respect to extra-articular gouty deposits.*

*• The double contour sign is the most sensitive finding for the assessment of gouty hand and wrist arthritis by US.*

*• Although the sensitivity of US for diagnosis of gouty hand and wrist arthritis is limited, it can be used as a first-line imaging modality in the presence of the DCS.*

## Introduction

The incidence of gout has tripled over recent decades and now represents the most common inflammatory arthritis in men and women [1–4].

Long-term hyperuricemia results in deposition of monosodium urate (MSU) crystals in joints and soft tissues, triggering gouty arthritis and, if not properly treated, the formation of gouty tophi [5–8]. With respect to imaging methods Bongartz et al. [4] showed that DECT has a significant impact on clinical decision-making when gout is suspected. In the studies by Dalbeth [9] and Zhang et al. [10], DECT is described as an advanced imaging method for longitudinal follow-up to monitor treatment response when looking at tophus size regression [11–13].

Few studies have evaluated the feasibility of ultrasound (US) as compared to dual-energy CT (DECT), which can differentiate calcium-rich material (high attenuation) from MSU crystal-rich material (low attenuation) for the diagnosis of gout [14–16]. Manger et al. [17] described periarticular gouty deposits in 9/12 hands and wrists, whereas Huppertz et al. [18] presented overall results for multiple joints (knees, feet, elbows and hands) without detailed sonographic results for hands and wrists in their patient-based evaluation.

Therefore the aim of our study was to evaluate US findings compared to DECT in terms of intra- and extra-articular gouty deposits in patients presenting with acute arthritis of the hand and/or wrist with suspected gout.

## Materials and methods

### Patients

This prospective, HIPPA-compliant study received institutional review board approval, and written informed consent was obtained from all participants.

Between 2015 and 2016, 180 patients with a history of gout fulfilling the American Rheumatism Association classification criteria [19] for gouty arthritis of the hand and/or wrist were included. The initial diagnosis was based on clinical symptoms including swelling of the wrist/hand and/or hotness, redness or tenderness for 1–4 weeks’ duration, as well as serum urate levels. Patients were referred to DECT and US within 1–3 days by the rheumatology department, after being examined by a rheumatologist with 15 years of experience (ASK) [19].

A single symptomatic hand/wrist was evaluated for each study participant. Patients with history of inflammatory rheumatic disease, hand/wrist trauma or previous hand/wrist surgery were excluded.

### DECT examination

All patients were referred to dual-source CT scan (Somatom Definition Flash; Siemens Healthineers) using two different energy levels (80 and 140 kV), with a previously described dual-tube protocol [14].

Scan parameters included a 2 × 64 × 0.625 mm acquisition at a rotation time of 0.33 s. Transverse sections were reconstructed from the DE data sets, at a resolution of 0.4 mm using the soft tissue kernel (D30) and bone kernel (B60). Tube currents ranged from 100 to 140 mAs for tube A and between 200 to 250 mAs for tube B, based upon automatic care dose CT software. The soft tissue kernel data sets of both tubes were loaded onto a Syngo Multi-Modality Workplace (Siemens Healthineers) and reconstructed with a commercially available software program (DE Gout; Siemens Healthineers). This software provides color-coded images in the transverse, sagittal and coronal image planes, with a slice thickness of 0.75 mm at a slice increment of 0.5 mm. The CT examination extended from the distal forearm to the fingertips.

A commercially available picture archiving and communication system (PACS) was used (IMPAX; Agfa-Gevaert).

Color-coded DECT images were evaluated by a radiologist with 5 years of experience (SS), who was blinded to clinical data and US examination and who classified the findings as positive or negative for the presence of MSU deposits. A positive scan was defined as the presence of color-coded MSU deposits according to Huppertz et al. [18]. In addition, we included a detailed assessment of the location of MSU deposits to differentiate intra- and extra-articular deposits as follows:

Intra-articular: triangular fibrocartilage complex, distal radioulnar joint, radiocarpal joint, intercarpal, carpometacarpal joint, metacarpal joint, proximal interphalangeal joint, distal interphalangeal joint, erosion, and double contour sign. Extra-articular: extensor and flexor tendons, and lymphedema.

According to American College of Rheumatology (ACR)/European League Against Rheumatism (EULAR) guidelines nail bed deposits, sub-millimetre deposits, skin deposits, and deposits obscured by motion, beam hardening and vascular artefact were not classified as positive findings in our study [20].

### US examination

Sonographic examination was performed according to European Society of Musculoskeletal Radiology (ESSR) guidelines, using a 15–6 MHz linear array transducer (HI Vision Preirus, Hitachi Aloka Medical) by a musculoskeletal radiologist with 10 years of experience (ASK), blinded to DECT and clinical findings and not allowed to talk to the patient regarding further details.

A positive US diagnosis was provided in the presence of a double contour sign (DCS) or based upon visible aggregates or tophi and tendon depositions according to international consensus OMERACT (outcome measures in rheumatoid arthritis clinical trials) guidelines [21].

The DCS was characterized by an abnormal hyperechoic band over the superficial margin of the articular hyaline cartilage, independent of the angle of insonation. This band might be irregular or regular, continuous or intermittent, and could be distinguished from the cartilage interface sign and from CPPD deposits located within the cartilage [21].

A tophus was a circumscribed, inhomogeneous, hyperechoic and/or hypoechoic aggregation (which may or may not generate posterior acoustic shadow), which might be surrounded by a small anechoic rim [21].

Aggregates (which might be intra-articular or intratendinous) were heterogeneous hyperechoic foci that maintained a high degree of reflectivity even when the gain setting was minimized or the insonation angle was changed and which occasionally generated posterior acoustic shadow [21].

Intra-articular US findings included DCS, aggregates, tophi and erosions. For extra-articular US findings extensor and flexor tendons were evaluated.

Absence of the above described findings was tabulated as a negative US study, while any detected finding that did not match with the typical criteria of OMERACT guidelines was considered indeterminate (e.g. interrupted or very thin double contour line or hyperechoic unclear deposits, which might represent calcifications in tendons or might be caused by osteoarthritis). Indeterminate US findings were tabulated as missed by US relative to DECT.

### Clinical parameters

Clinical assessment was performed in patients presenting with acute hand/wrist arthritis having a history of gout based on the American Rheumatism Association classification criteria [19]. Serum uric acid levels and CRP were determine at the time of US and DECT examination according to ACR/EULAR guidelines and were rated respectively as elevated above 6 mg/dl [20] and CRP above 0.5 mg/dl. Both DECT and US operators were blinded to clinical and laboratory findings, as well as to each other’s imaging findings.

### Statistical analysis

Statistical analysis was performed with Stata 12.1 (StataCorp). The presence of positive findings for gout was tabulated for each location in the hand/wrist for each DECT and US. DECT and US findings were observed and recorded independently. The sensitivity and specificity of US were calculated with DECT as the gold standard. Since the US and DECT studies were performed on the same patients (paired study design), the frequency of positive findings for gout based upon US versus DECT was compared with a McNemar’s chi square for symmetry, with a *p* value of 0.05 indicating a statistically significant result. Three comparisons of frequency of positive findings for gout were performed between DECT and US: frequency of overall diagnosis, frequency of extra-articular diagnosis and frequency of intra-articular diagnosis.

## Results

### DECT and US findings

An overall diagnosis of gout (intra-articular and/or extra-articular) in 180 patients (136 men and 44 women, mean age, 65.9 years; age range, 31–93 years, disease duration 1–9 years, mean 5 ± 3.4 years) was demonstrated in 71/180 (39.4%) of patients by US, and in 97/180 (53.9 %) of patients by DECT (McNemar chi square for symmetry, *p* < 0.0001). Among the 83 patients with a negative DECT study, the final alternative diagnosis was confirmed as osteoarthritis in 42/83 (50.6%), calcium pyrophosphate dehydrate deposition disease (CPPD) in 31/83 (37.3%) and hydroxyapatite deposition disease (HADD) in 10/83 (12%). DECT and US findings were concordant for the overall diagnosis of gouty arthritis in 110/180 (61.1%) of patients (Table 1).

**Table 1:** Correlation between US and DECT

			US			
			1	2	3	Total
	Total	1	42	3	38	
		2	20	68	9	
DECT	Intra-articular	1	58	8	38	104
		2	9	61	6	76
		Total	67	69	44	180
	Extra-articular	1	94	4	9	107
		2	42	31	0	73
		Total	136	35	9	180

*1* negative findings, *2* positive findings, *3* indeterminate findings, *US* ultrasound,

*DECT* dual-energy computed tomography

Among 97 patients with a positive diagnosis of gout by DECT, US was positive in 68/97 patients (sensitivity = 70.1%). Among 83 patients with a negative DECT study, US was negative in 42/83 (specificity = 51%). When indeterminate US studies were counted as negative, the specificity of US increased to 96.4% (80/83).

**Fig. 1:**
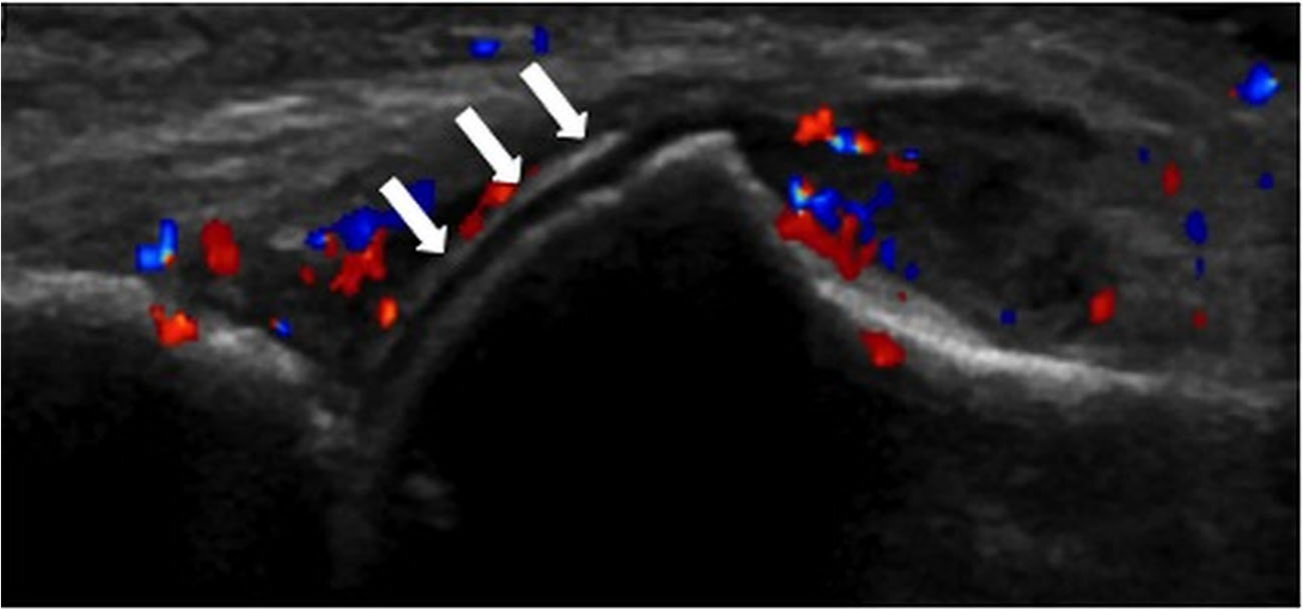
Right hand of a 77-year-old male patient. Longitudinal ultrasound (US) scan of metacarpophalangeal (MCP) joint showing double contour sign (arrows). Neither tophi nor aggregates can be seen. Dual-energy CT (DECT) of the same joint (not shown) was negative

**Fig. 2:**
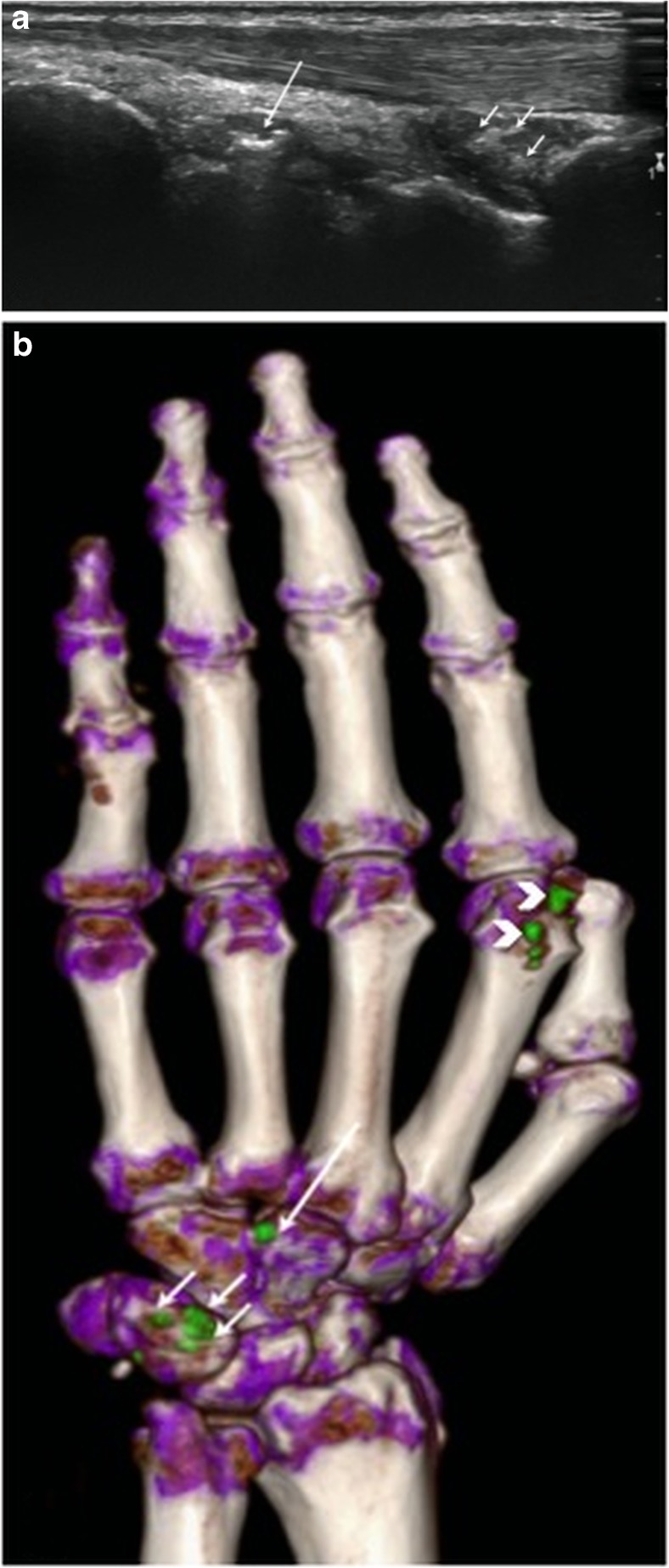
Left hand of a 56-year-old female patient. a Longitudinal US scan over the dorsal wrist shows small echogenic deposits in the radiocarpal joint (short arrows) and intercarpal joint (large arrow), but no typical aggregates or tophi and was rated as indeterminate case. b DECT 3D volume rendered image of the same hand showing MSU deposits at the radiocarpal joint (short arrows) and intercarpal joint (large arrow). Note: MSU deposits at dorsal aspect of 2nd MCP joint (small white arrow heads)

**Fig. 3:**
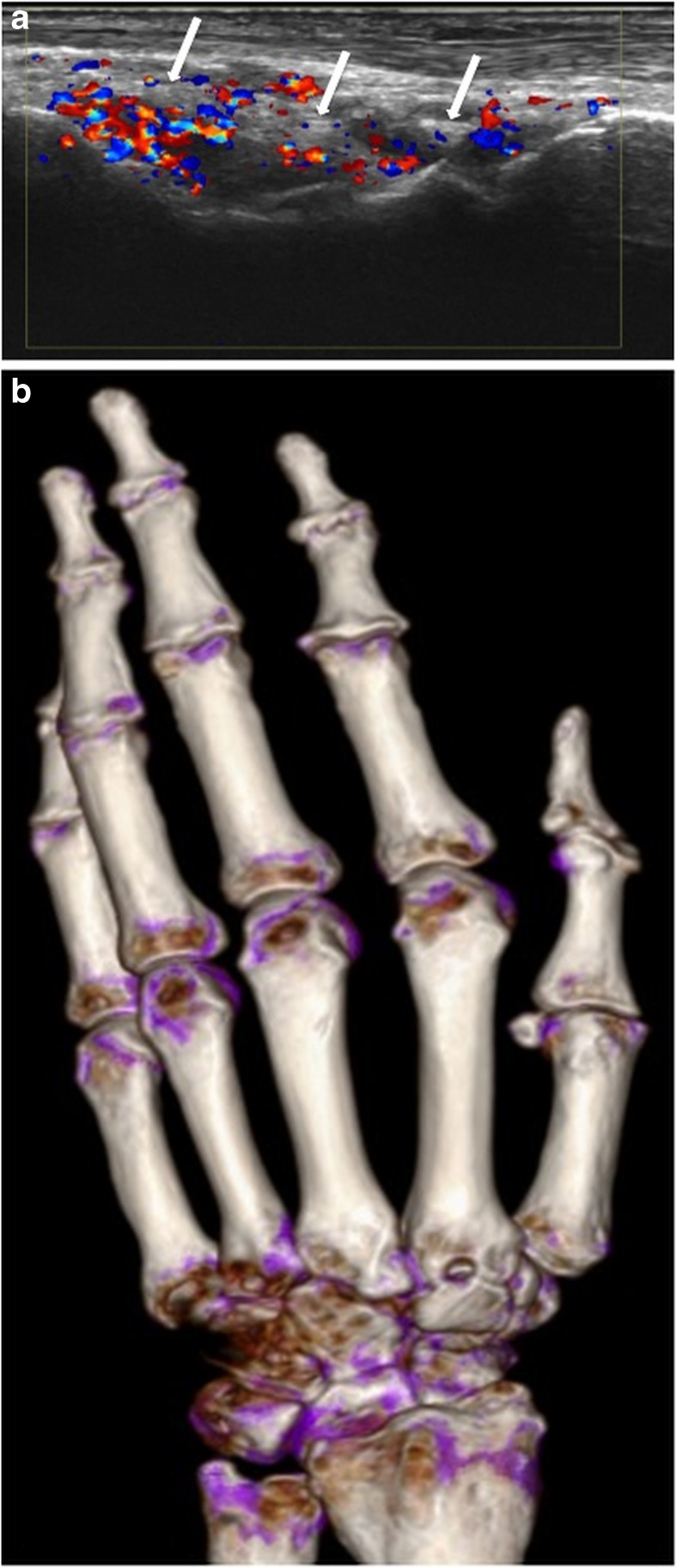
Left hand of a 63-year-old male patient. a Longitudinal US scan of the dorsal wrist showing hypoechoic thickening, extensive hyperemia and echogenic deposits (white arrows) in RC and IC joints, rated as indeterminate. b DECT 3D volume rendered image of the same hand showing no MSU deposits, but severe osteoarthritis at RC, IC and carpometacarpal joints in terms of osteoarthritis

The distribution of US and DECT findings are summarized in Table 2. A comparison between US and DECT is provided for intra- and extra-articular findings in Table 1. Among 73 patients with positive extra-articular findings on DECT, US was positive for an extra-articular finding in 31 patients (sensitivity = 42.5%, McNemar chi square for symmetry, *p* < 0.0001). Among 76 patients with positive intra-articular findings on DECT, US was positive for an intra-articular finding in 61 patients (sensitivity = 80.3%, McNemar chi square for symmetry, *p* = 0.14). The DCS was the most frequently positive US sign for the diagnosis of gouty hand and wrist arthritis (Figs. 1, 2 and 3). Among the 61 patients with a positive US study for intra-articular gout that was concordant with DECT, DCS was present in 58 (95.1%). Overall sensitivity of the DCS among the 97 patients with a positive DECT study was 63/97 (sensitivity = 65%), and among the 76 patients with a positive intra-articular DECT study was 58/76 (sensitivity = 76%). A positive DCS was also present in 3/83 patients with a negative DECT study (specificity = 96.4%). Thus, 63/66 patients with a DCS had MSU deposits on DECT.

**Table 2:** Comparison of deposition sites between DECT and US

	Deposition site	DECT Number of patients	%	US Number of patients	%
Intra-articular	TFCC/DRU	53/97	54.6	34/97	35.1
	RC	53/97	54.6	34/97	35.1
	IC	55/97	56.7	34/97	35.1
	CMC	34/97	35.1	29/97	29.9
	MCP	41/97	42.3	41/97	42.3
	PIP	32/97	33	20/97	20.6
	DIP	16/97	16.5	11/97	11.3
	DCS	–	–	66/97	68
Extra-articular	Extensor tendons	29/97	30	16/97	16.5
	Flexor tendons	63/97	65	11/97	11.3

*TFCC/DRU* triangular fibrocartilage complex/distal radioulnar joint, *RC* radiocarpal joint, *IC* intercarpal joint, *CMC* carpometacarpal joint, *MCP* metacarpophalangeal joint, *PIP* proximal interphalangeal joint, *DIP* distal interphalangeal joint

### Serum uric acid level

Mean uric acid level in the study population was 7.07 mg/dl (range 1.89–15.83 mg/dl, SD ± 4.01 mg/dl) and CRP was 4.5 ± 0.7 mg/dl (range 0–0.5 mg/dl). An elevated serum uric acid was present in 71/180 (39.4%) patients.

Mean uric acid levels were only marginally higher among patients with a positive DECT study and a final diagnosis of gout, as compared to those with a negative DECT study and an alternative confirmed diagnosis (7.4 mg/dl versus 6.6 mg/dl), *p =* 0.06.

Patients with a positive US study for the diagnosis of gout did demonstrate a significantly higher uric acid level than those with a negative US study (7.7 mg/dl versus 6.2 mg/dl), *p* < 0.01. Uric acid levels were higher among patients with a positive DCS compared to those without DCS (7.7 mg/dl versus 6.7 mg/dl), *p* = 0.01.

## Discussion

**Fig. 4:**
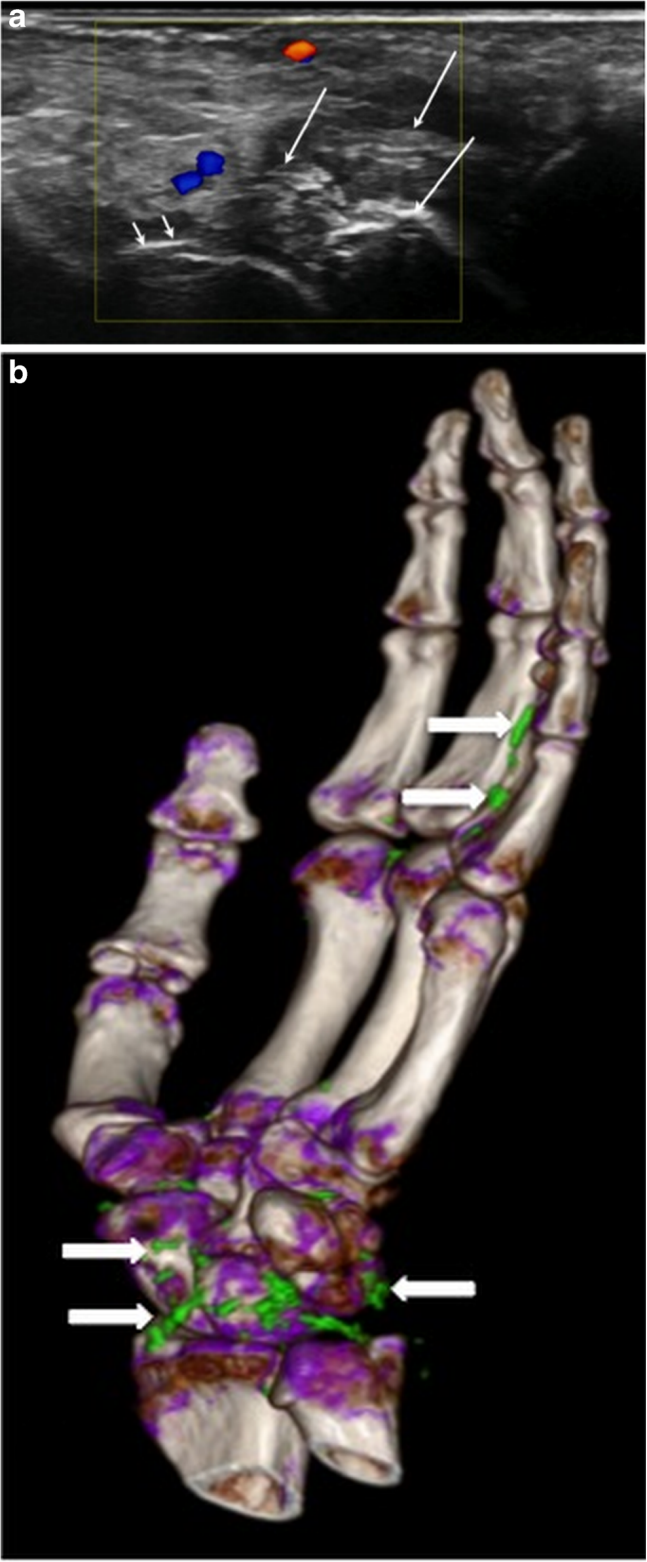
Right hand of a 63-year-old male patient. a Longitudinal US scan over the volar wrist showing tophus formation (large arrows) and double contour (small arrows). b DECT 3D volume rendered image of the same hand showing MSU deposits at the wrist and flexor tendons

The DCS was the most frequently detected US sign for the diagnosis of gouty hand and wrist arthritis with a sensitivity of 76% for intra-articular gout, with an overall sensitivity of 65% (Fig. 4). For the extra-articular compartment in terms of gouty deposits in tendons DECT was superior for delineation and characterization [22]. Our study is the first to give a detailed description of MSU depositions in both the intra- and extra-articular compartments of the hands and wrists with comparison of US to DECT.

US has been advanced as an imaging modality of choice for the diagnosis and management of gout [21, 23–25]. In fact, in addition to a positive DECT result, the DCS has been incorporated in ACR/EULAR guidelines 2015 as being the most specific sign for the diagnosis of gouty arthritis [24, 26].

Among patients with a positive DCS, a wide range of sensitivity for DCS has been reported, even for experienced sonographers [26–30]. However, a positive DCS and a negative DECT study might be explained also by a low concentration of MSU crystals or early disease with gouty deposits not yet visible on DECT. Thiele and Schlesinger [28] detected the DCS in 92% of 23 patients, but that study included primarily the first MTP joint and knees and only four MCP joints. Ottaviani et al. [31] reported a sensitivity of 57% and specificity of 98% for DCS, but again with MTP joints and knees. Ogdie et al. [32] reported a pooled sensitivity for DCS of 0.83 (0.72–0.91) and specificity of 0.76 (0.68–0.83) in a literature review of 11 studies. A recent study by Naredo et al. [33] showed a sensitivity of 84.6% and specificity of 83.3% for DCS in upper and lower limbs in a 12-structure US assessment. On the basis of these studies, the DCS has been incorporated into the ACR/EULAR guidelines [32].

As detailed in our results, 63 of the 66 patients with a positive DCS had evidence of MSU deposits on DECT. A phantom study by Diekhoff et al. [34] demonstrated that gouty deposits can reliably be detected by DECT at a concentration of 12.5%, and CPPD at a concentration of 6.25%, corresponding to deposits with mean Hounsfield unit values of 59.8 for MSU and 101.1 for CPPD [29]. For in vivo assessment the estimated lower limit of uric acid concentration per voxel has to be 35% or higher to be detectable by DECT [4, 32, 35, 36]. It is possible that the three cases of positive DCS in our study with negative DECT findings may represent low concentration MSU disease or early disease with gouty deposits not yet visible on DECT. Furthermore, the presence of a DCS in our study population was related to an elevated mean uric acid level. Careful follow-up for further signs of gouty arthritis both clinically and by US is planned for these three subjects.

As recorded in our series, only 71/180 patients (39.4%) had a definitive diagnosis of gout with US versus 97/180 patients (53.9%) by DECT. Indeterminate US cases frequently had small deposits, suggesting that cases with less severe findings may be more challenging. Our study shows that osteoarthritis (OA) can be challenging in the US differential diagnosis, as 50.6% of the DECT negative patients were diagnosed to have OA with loose bodies by using coronal reformed CT images, and many of these patients were positive or indeterminate by US. US shows echogenic findings which may be secondary to calcium or MSU deposits, while DECT is capable of discriminating urate from non-urate compounds [22] (Figs. 3 and 4). This limitation of US was confirmed in our study (Table 2) as US showed fewer extra-articular gouty deposits (extensor tendon 22.5%, flexor tendon 15.5%) versus DECT (extensor tendon 30%, flexor tendon 65%). A prospective blinded randomized study by Choi et al. [35] showed a sensitivity of 84% and a specificity of 93% for detection of MSU deposits by DECT, but the most common location of MSU deposits in 20 evaluated patients were in lower extremities, and included only 6 hands/wrists out of 40 joints. The role of DECT has also been shown by Hu et al. [37] reporting a high sensitivity and specificity of 75.2% and 92.7% of DECT for the detection of gouty arthritis in upper and lower extremities. Zhu et al. [38] compared the diagnostic accuracy of DECT and US in various joints in 40 patients, also showing that DECT was superior to US in upper limbs, whereas no difference between the two methods was seen for the lower limbs. Finkenstaedt et al. [11] reported a change in the treatment plan in 23/43 patients with DECT, including 37 hands/wrists in their study.

Our study has several limitations. A major limitation of our study is that synovial fluid (SF) aspiration was not performed, because feasibility is limited in clinical practise [14, 21, 22]. Given the small dimensions of the hand/wrist joint recesses, fluid aspirations with small amounts of joint effusion often fail and are painful for the patients. Aspiration of MSU crystals may fail due to periarticular crystal depositions, or due to sedimentation of MSU with aspiration of crystal-free SF. Bongartz et al. [4] showed that in several patients with a negative SF analysis, DECT demonstrated evidence of MSU deposition in tendon sheaths and enthesial sites, suggesting that a significant number of patients could be tested false negative by SF examination alone [36]. Glazebrook et al. reported a sensitivity of 100% for DECT and a specificity of 79% and 89% (in two readers) for DECT compared to MSU positive joint aspiration results, but only 43 patients were evaluated and only 14 patients showed MSU deposits in wrists [16, 36]. Furthermore SF aspiration may not be possible if the patient refuses or is on anticoagulation. Given the difficulty of using SF analysis as a gold standard, US and DECT assume greater importance.

The evaluation of US and DECT by a single radiologist precludes our ability to evaluate inter-observer variations. Finally, we included patients with short duration of gout or a lesser degree of hyperuricemia, which may lead to MSU crystal deposition below the threshold of detection of DECT. The limit of detection of DECT is generally considered to be 2 mm, so microscopic tophi may be missed [36]. A further limitation of our study is the high number of indeterminate cases by US (47 patients) limiting our specificity to 51%, increasing to 96.4% when indeterminate cases are counted as negative. These cases seem particularly to benefit from DECT evaluation and should be addressed in further studies.

In summary the percentage of gouty deposits detected by US was significantly lower than that by DECT, especially in the extra-articular spaces.

Although the sensitivity of US for diagnosis of gouty hand and wrist arthritis is limited, it can be used as a first-line imaging modality in the presence of the DCS.

DECT was superior on overall detection and characterization, which might be of importance not only for diagnosis but for therapeutic follow-up in terms of delineation of gouty burden.

## Abbreviations

CPPD: calcium pyrophosphate dehydrate deposition disease
DCS: double contour sign
DECT: dual-energy computed tomography
HADD: hydroxyapatite deposition disease
MSU: monosodium urate
SF: synovial fluid
US: ultrasound
